# Investigation of Depression and Post-Traumatic Growth in Renal Transplant Recipients via Self-Assessment

**DOI:** 10.3390/jpm14090999

**Published:** 2024-09-20

**Authors:** Zuleyha Simsek Yaban, Semra Bulbuloglu

**Affiliations:** 1 Division of Surgical Nursing, Department of Nursing, Faculty of Health Sciences, Kocaeli University, Kocaeli 41380, Turkey; zuleyha.simsek@kocaeli.edu.tr; 2 Division of Surgical Nursing, Department of Nursing, Faculty of Health Sciences, Istanbul Aydin University, Istanbul 34295, Turkey

**Keywords:** depression, renal transplant recipients, post-traumatic growth, recipients, renal transplant

## Abstract

**Objective:** In this study, we aimed to determine post-traumatic growth and depression levels in renal transplant recipients and the relationship between these two variables. **Design and Methods:** The study was conducted with a descriptive, cross-sectional, and correlational design. The data for the study were collected at the organ transplant unit of a research and training hospital located in the west of Turkey. The sample of the study included 122 kidney transplant recipients (n = 122). A Sociodemographic Information Form, the Post-Traumatic Growth (PTG) Inventory, and the Beck Depression Inventory (BDI) were employed to collect data. In the analyses of the data, descriptive statistics, ANOVA, an independent-samples *t*-test, post hoc tests, and Pearson correlation tests were used. **Results:** As the ages of the renal transplant recipients increased, their depression scores decreased, while their PTG scores increased. Higher depression levels were identified in the female participants compared to the male participants and in those with a low income compared to other income groups. The lowest PTG levels were found in the recipients who received their kidney transplants from third-degree relatives. Age, gender, economic status, and time of transplant were predictors of depression. The identity of the donor was the most significant predictor of PTG (62% explanation rate). A strong and inverse correlation was found between depression and PTG (*p* < 0.05). **Conclusions:** Post-traumatic growth was found to decrease depression. However, while poor economic status led to depression, high economic status did not lead to a significant change in PTG. As education levels increased, PTG decreased, but education status did not have any significant effect on depression. On the other hand, there was a negative correlation between PTG and depression. The results obtained in this study are valuable and important in terms of understanding depression better and determining PTG as a significant factor that could alleviate it.

## 1. Introduction

In chronic renal failure cases where all conservative treatment options are insufficient, renal transplant can be a life-saving surgical treatment option [1,2]. Difficulties experienced, starting from the first stage of chronic renal failure, continue in the kidney transplant process and in the short and long term in the postoperative period. All health experiences of kidney transplant recipients are rather stressful and require physical and psychological resilience [3]. Renal transplant recipients try to cope with the problems they experience in the perioperative period, and they are expected to struggle with these problems in the best way possible. It is generally thought that all problems will be solved after a renal transplant, but various care and treatment needs continue to be a problem. Most of these problems involve intra-psychological conflicts until they result in a traumatic experience for the individual and cause an existential crisis to emerge [4]. These conflicts and crises mostly result in depression. Depression is a significant cause of suicide [5] and disability [6] worldwide. There may be various social and psychological factors in the development of depression, while it is also thought that a functional polymorphism in the Xp11.4-Xp11.3 gene that encodes monoamine oxidases (MAOs) is associated with depression [7]. It was stated that elevated MAO-A levels measured in the prefrontal cortex and anterior cingulate cortex play an effective role in the emergence and exacerbation of depression symptoms [8,9]. The Diagnostic and Statistical Manual of Mental Disorders [DSM]-V defines two different forms of depression, “depressive disorders” and “bipolar disorders and related disorders” [10]. According to the results of previous studies, the development of depression in recipients after renal transplants reduces their adherence to immunosuppressive treatment and increases their morbidity rates [3,11,12,13]. In a study conducted with the participation of 260 renal transplant recipients, it was reported that almost all recipients had depressive symptoms, and depression disrupted treatment compliance [3], while in another study, it was stated that 86% of renal transplant recipients experienced low spirits and negative emotions [11].

In the study conducted by Jindal et al., the presence of depressive disorders prior to renal transplantation was not checked. Although this could be considered a limitation of the study, the exposure of renal transplant recipients to depression at any stage of the perioperative period is highly risky in that it can prevent them from adhering to their treatment and care sufficiently. To focus on their treatment and adhere to their care, it is highly important for renal transplant recipients to act carefully and believe that they will recover [12]. The rate of mortality in renal transplant recipients after transplant surgery was reported by Novak et al. as 21% in those with depressive symptoms and 15% in those without depressive symptoms. This dramatic result suggested that depression may be defined as a predictor of mortality because it was predicted that depression triggered cardiovascular diseases [13]. Uyar stated that after renal transplant surgery, depression disrupted the adherence of recipients to their immunosuppressive treatment, while the adverse effects caused by immunosuppressive treatment also led to depressive symptoms [3]. Frequently used immunosuppressive drugs are calcineurin inhibitors, antimetabolites, steroids, and mTOR inhibitors. These drugs have been associated with several minor and major complications, including hypertension, anemia, leukopenia, infection, vascular problems, hyperlipidemia, diarrhea, nausea–vomiting, and others [14,15,16].

It is possible for those who suffer psychologically in the aftermath of traumatic events to heal and their pains to transform into positive experiences, and they can end up adopting a positive mood that enables them to find meaning in life [17]. Post-traumatic growth (PTG) is a counter response that emerges when individuals are exposed to high stress and crisis [18]. Until this counter response develops, renal transplant recipients are likely to experience the fear of death countless times. Yet, when PTG starts, their coping mechanisms grow stronger, and their psychological resilience increases [19]. In this context, PTG represents an acquired ability and a mood in which an orientation towards the opposite of depressive thoughts and psychological resilience starts to grow and settles in the brain. In this sense, it is thought that there is an inverse relationship between depression and PTG. The depressive symptoms of individuals whose PTG strengthens can be alleviated. PTG can be added to non-pharmacological treatment methods in reducing depression. In this study, the aim was to analyze the depression and PTG levels of renal transplant recipients. The research questions of this study were as follows: “What are the depression levels of renal transplant recipients?”, “What are the PTG levels of renal transplant recipients?”, and “Is there a relationship between the depression levels and PTG levels of renal transplant recipients?”.

## 2. Materials and Methods

### 2.1. Study Design and Participants

This was a descriptive and cross-sectional study. The data were collected, prospectively, at the organ transplant unit of a research and training hospital located in the west of Turkey between 1 November 2022 and 31 May 2023. Renal transplant recipients who volunteered to participate in the study filled out the data collection forms in approximately 30 min. The sample size required to conduct the study was determined with 0.95 power and α = 0.05 margin of error. According to the power analysis results, the minimum required sample size was 94 participants (n = 94). The sample consisted of 122 renal transplant recipients. The inclusion and exclusion criteria of the study are presented below.

### 2.2. Inclusion and Exclusion Criteria

The inclusion criteria consisted of (i) being older than 18 years old, (ii) having no communication and language barriers, (iii) not being diagnosed with a psychiatric or psychological disease, and (iv) volunteering to participate in the study. The exclusion criteria were the opposite of the conditions specified in the inclusion criteria, as well as being patients who were still hospitalized.

### 2.3. Data Collection Method

In the collection of the data, the face-to-face interview and questionnaire methods were used. The data were collected by the researcher.

### 2.4. Data Collection Tools

The data were collected with a Sociodemographic Information Form, the Beck Depression Inventory, and the PTG Inventory. Information about the data collection tools is presented below.

### 2.5. Sociodemographic Information Form

The form included questions inquiring about the individual characteristics of the participants, such as age, gender, marital status, educational status, and economic status. There were also questions on their characteristics related to renal transplant surgery.

### 2.6. Beck Depression Inventory

The Beck Depression Inventory [BDI] is a 21-item scale developed in 1961 by Beck et al. [20]. It was adapted to Turkish by Hisli in 1988 [21]. There are many parameters evaluated on the BDI, such as the inability to experience pleasure, depression, low spirits, and sleep problems. The respondent chooses one of the four sentences formed to indicate their psychological status at that moment. Each item is scored between 0 and 3. The total score of the BDI ranges between 0 and 63. A score of seventeen points or above indicates the presence of depression. In the scale adaptation study conducted by Hisli, Cronbach’s alpha coefficient of the scale was reported as 0.80. In this study, this value was found to be 0.81.

### 2.7. Post-Traumatic Growth Inventory

The Post-Traumatic Growth Inventory [PTGI] is a self-report scale developed by Tedeschi and Calhoun in 1996 [22]. The 6-point Likert-type scale consists of 21 items. The Turkish adaptation study of the scale was conducted by Kagan et al. The scale score ranges between 0 and 105 [23]. Higher scale scores show that the individual has experienced more post-traumatic growth. The PTGI has three subscales, which are change in self-perception, change in relations, and change in philosophy of life. Cronbach’s alpha internal consistency coefficients of the subscales of the PTGI were reported to be 0.88, 0.77, and 0.78, respectively [23]. In this study, Cronbach’s alpha coefficient of the scale was determined as 0.88.

### 2.8. Statistical Analysis

In the analysis of the collected data, the IBM Statistical Package for the Social Sciences 25.0 (Armonk, NY, USA) was used. Before the analyses of the data, the normality of the distribution of the data was tested using the Kolmogorov–Smirnov test. Descriptive statistics (frequency, percentage, mean, and standard deviation) were used in the analyses of the data. To compare the variables, an independent-samples *t*-test and a one-way ANOVA were employed. Post hoc tests were performed to determine the sources of significant differences identified in previous tests. Additionally, Pearson’s correlation analysis was performed to identify the relationships between the scale scores of the participants. The results of the analyses were evaluated in a 95% confidence interval and at a statistical significance level of *p* < 0.05.

### 2.9. Ethical Aspect of the Study

Prior to the study, ethical approval was obtained from Istanbul University Cerrahpaşa Rectorate Clinical Research Ethics Committee (Date: 11 May 2022, Decision No: 2022/60), and institutional permission was received from Istanbul University Cerrahpaşa Research and Training Hospital’s Chief Physician (Institutional Review Board). The principles of the Declaration of Helsinki were observed at all stages of the study, and written consent was obtained from the renal transplant recipients who agreed to participate in the study. The recipients were included in the study after their written informed consent was obtained.

## 3. Results

The sociodemographic information of the participants is presented in Table 1. It was determined that 49.2% of the participants were female, 54.9% were married, and 39.3% had university or higher degrees. While 52.5% of the participants were employed, 45.1% had moderate levels of income. To determine the sources of score differences among more than two variables, post hoc tests were performed. According to the post hoc test results, as the ages of the participants increased, their PTG levels increased, while their depression levels decreased. As the educational levels of the participants increased, their PTG levels decreased. The highest levels of depression were found in the participants with low economic status.

The health-related information of the participants is outlined in Table 2. The participants who underwent renal transplantation 10–20 years ago constituted 41.7% of the sample. The donors of 61.5% of the participants were mothers, fathers, or siblings. Living donors were used by 81.1% of the participants. Cardiovascular problems were experienced by 25.4% of the participants, while 23.8% had gastrointestinal problems. As the duration after their renal transplant surgeries increased, the depressions levels of the participants decreased, and their PTG levels increased. The lowest PTG levels were determined in the participants who received kidneys from their third-degree relatives. The complication development statuses of the participants did not have a significant effect on their BDI and PTGI scores.

Table 3 presents the mean BDI and PTGI scores of the participants and the results of the correlation analysis conducted between these scores. The mean BDI and PTGI scores of the participants were determined to be 33.62 ± 9.26 and 84.45 ± 20.09, respectively. A strong, negative, and statistically significant correlation was found between the depression and PTG levels of the participants (r = −0.836, *p* = 0.012).

**Figure 1:**
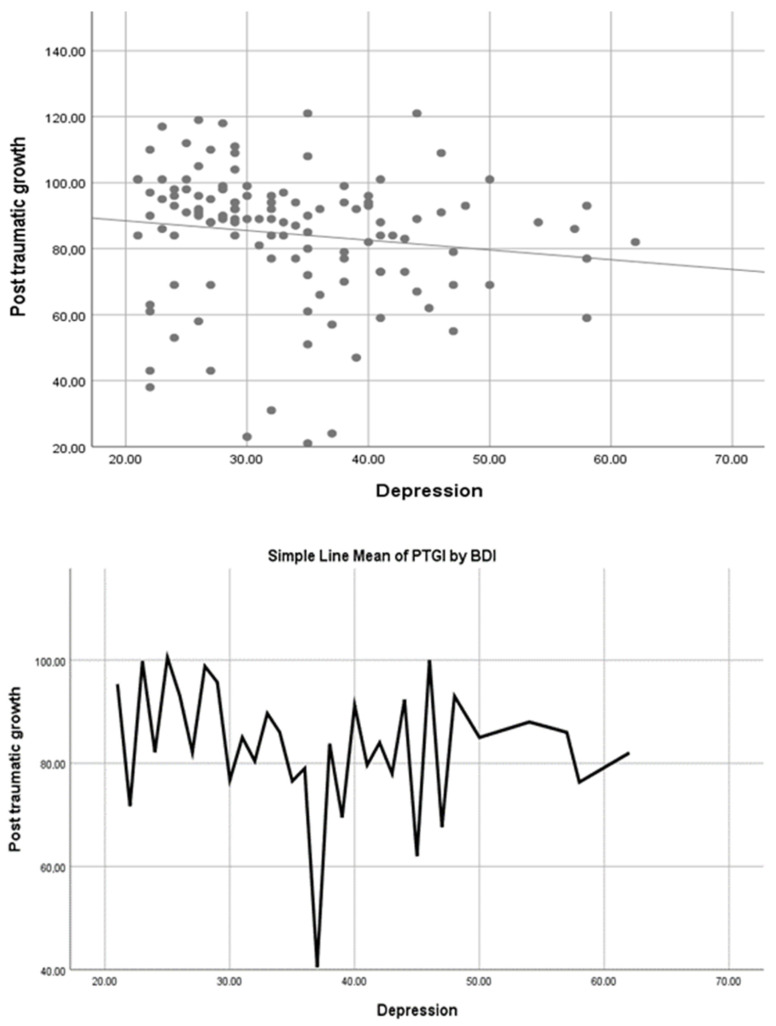
Comparisons of mean PTGI and BDI scores.

Figure 1 shows the comparison between the mean PTGI and BDI scores of the participants. Accordingly, there was an inverse relationship between the PTGI and BDI scores of the participants. It can be concluded that depression decreases as post-traumatic growth increases.

## 4. Discussion

Kidney transplantation is a rather stressful experience that requires a high level of psychological resilience. In this study, conducted with the participation of 122 renal transplant recipients, it was determined that all renal transplant recipients, who were going through quite challenging days, had moderate or higher levels of depressive disorder (cut-off point: 17). Following renal transplantation, recipients continue their lives by using multiple drugs, particularly immunosuppressive drugs [24]. There are studies in the literature which have demonstrated depressive symptoms in renal transplant recipients after transplant surgery [3,4,25,26,27,28]. It has been reported that the prevalence of depression in recipients after renal transplant surgery is very high and varies between 25% and 51.6% [4,25,26,27,28], while the severity of depression is usually above-average, which was in agreement with the result of our study [3]. However, prescribing antidepressants to this patient group means increasing their medication burden and the number of drugs they use. In fact, antidepressants are not used in the primary treatment of mild depression. In mild to moderate depression cases, psychological interventions are used as non-pharmacological treatment methods [29]. In the presence of severe depression, on the other hand, mindfulness and cognitive behavioral therapy methods should be used in combination with antidepressants [30]. Cognition-based therapies are therapeutic methods that are structured to change the negative thinking of patients and help them control their negative thoughts [31].

It is seen that individuals with depressive disorders mostly focus on the traumatic event that they have experienced [3]. In the PTG process, individuals believe that they will reach wisdom starting their journey from difficulties, and they focus on how to eliminate the negative results that develop in relation to the traumatic event [17]. PTG has been explained as the better functioning of individuals in life and their adoption of steps towards self-realization [32]. In this study, it was determined that PTG reduced depression, and the relationship between PTG and depression was strong, negative, and statistically significant. Additionally, certain social conditions that supported depression were found. Younger participants and the participants who underwent renal transplant surgery a short time ago were more depressed. Similarly, as age increased, and the time passed after transplant surgery increased, the PTG levels of the participants also increased. Increased PTG along with age can be related to personality maturation, and as the time passing after transplant surgery increases, the adjustment of the recipient may increase. It was also determined in this study that the PTG scores of the participants whose donors were their first-degree relatives, such as mothers, fathers, spouses, and siblings, were higher. This could be the result of the intense feeling of social support experienced by the participants. Social support was reported to increase PTG in liver transplant recipients [33]. The findings of this study were supported by the literature in this regard.

In the literature review, it was found that PTG was reported to increase healing [17] and psychological resilience and reduce stress [34] and depression [35]. No study in the literature in which the relationship between PTG and depression was examined in renal transplant recipients was encountered. Therefore, the results of this study were rather significant.

Interventions to prevent depression after renal transplant surgeries should involve integrative and holistic healthcare practices provided by teams that include physicians, nurses, psychologists, psychiatrists, and professionals from other disciplines [36,37,38,39]. By using educational techniques and therapeutic approaches, renal transplant recipients can be supported in terms of PTG. Considering the results obtained in this study, PTG can be added to other cognitive therapies as an effective non-pharmacological therapy method in the fight against depression. It should be noted that non-pharmacological therapy methods do not have any side effects. Moreover, although frequently used psychological interventions are useful in terms of quantity, quality, and effectiveness, there remains a need for numerous non-pharmacological therapy methods that could be effective in coping with depression, and PTG can be a candidate for a good therapeutic method.

This study had certain limitations: (i) The results cannot be generalized due to the small sample size. (ii) The study had a descriptive design, and there was no control group. A control group that consisted of renal transplant recipients whose PTG levels were low and who did not have depression could have been included in the study. Hence, by using BDI and PTGI and with the participation of renal transplant recipients, multi-center studies with larger sample sizes can be conducted. (iii) The depression levels and PTG levels of the renal transplant recipients included in the study could have been affected by various factors, and these factors (such as pathophysiological issues) might have been neglected. (iv) The researchers were present in evaluating the BDI responses of the participants, and this situation posed a risk of potential bias. (v) Vitamin D therapy can heal mental health, and its deficiency can exacerbate it. However, in this study, vitamin D use/deficiency was not evaluated.

## 5. Conclusions

In this study, an inverse correlation was found between PTG and depression in renal transplant recipients. This finding suggests that healthcare professionals should be sensitive to recognize depressive symptoms following renal transplant surgery and understand the importance of PTG. In addition to the relationship between depression and PTG, each had its own predictors. Poor economic status is a predictor leading to depression but is a changeable one. As time passed by after their transplant surgery, and the participants got older, their PTG levels increased. This finding points to a need for more psychological interventions in the early period after renal transplants. Good economic status did not increase the PTG of the participants, but the severity of depression was higher at younger ages. Experiences gained along with advancing age could have increased the inclination of the participants towards gaining PTG. All these results should be considered in psychological interventions to be made in individuals with depression.

## Figures and Tables

**Table 1:** Sociodemographic variables and BDI and PTGI scores of the renal transplant recipients.

Age ($\bar{X}$ ± Sd) (Min, Max)	41.09 ± 13.26 (18, 73)		PTGI	BDI
Descriptive Characteristics	n	%	$\bar{X}$ ± Sd	$\bar{X}$ ± Sd
Age				
18–35 (1)	45	36.9	78.53 ± 22.61	35.53 ± 10.98
36–45 (2)	38	31.1	85.93 ± 19.7	33.68 ± 8.41
46 or older (3)	39	32	88.76 ± 16.55	31.35 ± 7.41
Test and Sig.			F = 0.136, *p* = 0.012 *	F = 0.971, *p* = 0.036 *
Post hoc test			1 < 2 < 3	1 > 2 > 3
Gender				
Female	60	49.2	82.3 ± 23.25	36.73 ± 8.99
Male	62	50.8	86.53 ± 16.39	32.54 ± 9.46
Test and Sig.			t = 1.018, *p* = 654	t = 3.065, *p* = 0.111
Marital Status				
Married	67	54.9	82.2 ± 18.8	33 ± 8.79
Single	55	45.1	87.18 ± 21.41	34.38 ± 9.83
Test and Sig.			t = 0.123, *p* = 0.360	t = 0.434, *p* = 0.419
Education				
Primary School (1)	25	20.5	95.38 ± 28.34	32.77 ± 7.58
Secondary School (2)	14	11.5	87.54 ± 21.07	34.78 ± 7.95
High School (3)	35	28.7	84.71 ± 18.27	31.95 ± 11.38
University or above (4)	48	39.3	81.47 ± 18.04	33.62 ± 9.26
Test and Sig.			F = 1.928, *p* = 0.000 **	F = 9.873, *p* = 0.217
Post hoc test			1 > 2 > 3 > 4	
Employment Status				
Retired	20	16.4	80.36 ± 19.67	32.8 ± 7.6
Unemployed	38	31.1	83.47 ± 22.24	36.5 ± 11.16
Employed	64	52.5	85.78 ± 18.53	32.17 ± 8.17
Test and Sig.			F = 2.209, *p* = 0.999	F = 0.654, *p* = 0.057
Economic Status				
High (1)	24	19.7	84.37 ± 19.78	30.75 ± 8.4
Moderate (2)	55	45.1	84.92 ± 20.07	32.09 ± 8.38
Low (3)	43	35.2	83.76 ± 20.61	37.18 ± 9.87
Test and Sig			F = 0.313, *p* = 0.754	F = 0.211, *p* = 0.021 *
Post hoc test				3 > 1, 2

$\bar{X}$ ± Sd, mean and standard deviation; * *p* < 0.05, ** *p* < 0.01; F = one-way ANOVA; t = independent-samples *t*-test.

**Table 2:** The health-related information and BDI and PTGI scores of the renal transplant recipients.

Health-Related Characteristics	n	%	PTGI	BDI
			$\bar{X}$ ± Sd	$\bar{X}$ ± Sd
Time of Transplant				
Less than 1 year (1)	8	6.6	75.83 ± 13.32	37.5 ± 4.04
1–5 years (2)	29	23.8	87.80 ± 19.7	37.36 ± 8.09
5–10 years (3)	34	27.9	92.62 ± 12.16	27.56 ± 7.38
10–20 years (4)	51	41.7	94.80 ± 20.59	26.41 ± 6.51
Test and Sig.			F = 4.982, *p* = 0.023 *	F = 0.701, *p* = 0.001 **
Post hoc test			4 > 3 > 2 > 1	4 < 3 < 1, 2
Donor’s Identity				
Mother, father, or sibling (1)	75	61.5	86.51 ± 15.74	37.2 ± 19.68
Spouse or child (2)	17	13.9	87.13 ± 17.84	34 ± 9.69
Third-degree relative (3)	30	24.6	84 ± 16.29	34.14 ± 6.64
Test and Sig.			F = 1.159, *p* = 0.007 *	F = 0.321, *p* = 0.411
Post hoc			1, 2 > 3	
Donor Type				
Living	99	81.1	86.01 ± 18.17	33.89 ± 9.42
Cadaveric	23	18.9	77.73 ± 21.94	32.43 ± 8.62
Test and Sig.			t = 3.539, *p* = 0.792	t = 0.808, *p* = 0.528
Complications ^ˤ^				
Cardiovascular	31	25.4	87.03 ± 18.13	36.01 ± 7.99
Endocrine	18	14.8	86.58 ± 17.02	34.57 ± 8.56
Gastrointestinal	29	23.8	83.8 ± 20.09	33.56 ± 9.21
Neurological (headache—2 people, circadian rhythm disorder—1 person, tremor—1 person, peripheral neuropathy—1 person)	5	4	84.45 ± 19.39	32.74 ± 8.31
Respiratory	14	11.5	83.53 ± 18.44	33.62 ± 9.16
Hematologic	7	5.7	87.03 ± 16.96	33.71 ± 9.36
Other (cancer, skin, musculoskeletal, and urinary)	13	10.6	81.83 ± 19.54	31.83 ± 13.85
Test and Sig.			F = 2.045, *p* = 0.073	F = 0.081, *p* = 0.948

ˤ Multiple choices were allowed. $\bar{X}$ ± Sd, mean and standard deviation; * *p* < 0.05, ** *p* < 0.01; F = one-way ANOVA; t = independent-samples *t*-test.

**Table 3:** Mean BDI and PTGI scores of the recipients and results of the correlation analysis.

Total	Number of Items	Items	Score Range	Min.–Max.	$\bar{X}$ ± Sd	Pearson Correlation Analysis and Sig.
BDI	21	1–21	0–63	21–62	33.62 ± 9.26	r = −0.836, *p* = 0.012
PTGI	21	1–21	0–105	6–105	84.45 ± 20.09	

## Data Availability

The datasets generated and/or analyzed in this study are available from the corresponding author upon reasonable request.
